# Meningitis caused by oral anaerobes detected using mNGS tool: a case report and review of literature

**DOI:** 10.1186/s12883-023-03307-2

**Published:** 2023-09-30

**Authors:** Xiaoqing Zhong, Miaomiao Wang, Qingxia Meng, Xuebin Jiang, Zhendong Guo, Yunzhou Zhang, Daiquan Gao

**Affiliations:** 1 Department Internal Medicine, MengZhou Minsheng Hospital, Mengzhou City, 454750 Jiaozuo, Henan Province P.R. China; 2 Department of Neurology, Pingdingshan First People’s Hospital, Weidong District, Pingdingshan, 467021 Henan Province P.R. China; 3 Internal Medicine-Neurology Wuqiao people’s Hospital, Wuqiao County, Cangzhou, 061800 Hebei Province P.R. China; 4 Intensive Care Unit, Beijing Renhe Hospital, Daxing District, Beijing, 102600 P.R. China; 5 Department of Pulmonary and Critical Care Medicine, Cangzhou People’s Hospital, Xinhua District, Cangzhou, 061000 P.R. China; 6 Department of Neurology, Xuanwu Hospital, Capital Medical University, No.45 Changchun Street, Xicheng District, Beijing, 100032 P.R. China

**Keywords:** Bacterial meningitis, Oral anaerobes, Cerebrospinal fluid, Next-generation sequencing

## Abstract

**Background:**

Bacterial meningitis is a central nervous system (CNS) infection disease of the meninges and brain parenchyma caused by the bacteria. Few cases of meningitis related to oral anaerobes have been reported in the literature. Here, we report a case of meningitis in a middle-aged woman, caused by oral anaerobes.

**Case presentation:**

A 58-year-old woman was admitted to hospital with fever, headache for 21 days and left limb weakness for 2 days. The blood cell counts (11.73 × 10^9^/L), neutrophil counts (9.22 × 10^9^/L) and high-sensitivity C-reactive protein levels (> 5.00 mg/L) were elevated. The brain computerized tomography (CT) scanning indicated the new right thalamus infarct. The brain cranial-enhanced magnetic resonance imaging (MRI) showed the right lateral paraventricular and right thalamic infarct, and abnormal signal in occipital horns of bilateral lateral ventricles were increased. In addition, the brain enhanced nuclear magnetic resonance (NMR) scanning suggested that meninges were thickened and enhanced at the base of the brain, with meningitis changes. The neck CT angiography (CTA) revealed arteriosclerotic changes. The metagenomic next-generation sequencing (mNGS) revealed *Eubacterium brachy, Porphyromonas gingivalis, Fusobacterium nucleatum* and *Torque teno virus* in her cerebrospinal fluid (CSF). The patient was diagnosed with purulent meningitis caused by infection of oral anaerobes, and treated with mannitol, ceftriaxone and vancomycin. Her symptoms alleviated. Subsequently, she was transferred to the infectious department and treated with ceftriaxone plus metronidazole (anti-anaerobes) and mannitol (reduce intracranial pressure). Her symptoms improved and currently received rehabilitation treatment.

**Conclusion:**

We herein report a rare case involving meningitis caused by infection of oral anaerobes. The mNGS can accurately detect the pathogens of infectious diseases.

## Background

Bacterial meningitis is an acute purulent infection of the subarachnoid space with bacterial pathogens, resulting in inflammation of the brain linings, which cause significant morbidity and mortality worldwide [1, 2]. Bacterial meningitis is usually diagnosed via detecting cerebrospinal fluid (CSF). The headache, acute fever and neck stiffness are typical clinical features of bacterial meningitis [3]. Headache is the most common symptom (85%), followed by fever (80%), nausea and vomiting may also be present [4]. The most common bacteria causing meningitis, *Streptococcus pneumoniae* and *Neisseria meningitidis*, which initially colonize the nasopharynx by attaching to the nasopharyngeal epithelial cells [5]. *S. pneumoniae* infection in adults that are able to adhere to the endothelium of cerebral capillaries and pass through or between cells to invade the CSF [5]. However, bacterial meningitis in adult caused by anaerobes bacteria, especially oral anaerobes, is a rare condition.

As a significant fraction of the oral bacteriome, anaerobes play a crucial role in the formation of multi-species biofilms attached to various anatomical sites [6, 7]. Recently, several studies have demonstrated that oral anaerobes can not only cause oral infectious diseases, but also are closely related to infectious diseases of other organs. For instance, *Porphyromonas gingivalis* infection can cause brain abscesses [8], appendicitis [9] and osteomyelitis [10]. However, anaerobes are often overlooked and infrequently reported due to the difficulties involved in their isolation and identification. Fortunately, metagenomic next-generation sequencing (mNGS) has been reported to be an important diagnostic modality for identifying uncommon pathogens [11]. Herein, we report a rare case of meningitis caused by oral anaerobes detected using mNGS tool.

## Case presentation

A 58-year-old woman was admitted to the hospital with fever, headache for 21 days and left limb weakness for 2 days. The patient had chronic headache (30 years) and dental caries with intermittent toothache (6 years). Twenty-one days (Jun 20) before admission, the patient had fever, swelling pain on his forehead, cough and expectoration, then she was treated in a local clinic. Subsequently, the fever and headache symptoms aggravated (the maximum body temperature reached 38 °C), accompanied by nausea, vomiting, rash, night sweats and arthralgia. The patient was transferred to Beijing Shunyi hospital (Beijing, China), and treated with cefonicid (anti-infective) and amlodipine (reduced blood press), but the fever and headache continued. 2 days (Jul 9) before admission, the patient suddenly developed left-sided limb weakness (the strength grade of left-limb was IV stage) that did not affect standing or walking.

On Jul 11, the patient was transferred to our hospital. She exhibited somnolence, bilateral equal-sized round pupils (diameter 3 mm) and sensitivity to light. Her eyes movement in all directions were not limited, and there was no nystagmus, symmetric bilateral nasolabial folds, normal muscle strength in the four limbs (the strength grade of four limbs was V stage), and unstable ataxic movement. In addition, she had no obvious objective sensory disturbance (sensory plane) and the pathologic reflexes were not elicited. The sign of meningeal irritation (Kemig’ and Brudzinsk’ signs) and Brudzinski’s sign were positive. Laboratory test results revealed that the white blood cell counts (11.73 × 10^9^/L), neutrophil counts (9.22 × 10^9^/L) and high-sensitivity C-reactive protein levels (> 5.00 mg/L) were elevated, the level of procalcitonin (PCT) was < 0.1 ng/mL, and the autoimmune encephalitis antibodies (AMPAR1-Ab, AMPAR2-Ab, CASPR2-Ab, DPPX-Ab, GABABR-Ab, IgLON5–Ab, LGI1-Ab, NMDAR-Ab), paraneoplastic antibodies (anti-Amphiphysin, anti-CV2, anti-GAD65, anti-Hu, anti-PNMA2 (Ma-2/Ta), anti-Recoverin, anti-Ri, anti-SOX1, anti-Titin, anti-Tr (DNER), anti-Yo, anti-Zic4 ) and demyelinating antibodies (AQP4-Ab, GFAP-Ab, MBP-Ab, MOG-Ab) were negative. On Jul 12, a lumbar puncture was performed and the test results were shown in Table 1. The mNGS (MBX52313, Tianjin Genskey Medical Technology Co., Ltd, Tianjing, China) results showed that the *Eubacterium brachy, P. gingivalis, Fusobacterium nucleatum* and *Torque teno virus 29* were detected, but the fungi and mycobacterium tuberculosis were not detected.

**Table 1:** The serum findings

Projects	Values
CSF pressure	> 330 mm H_2_O
Total cellular score	21,302 × 10^6^/L
Erythrocyte	16,000 × 10^6^/L
White blood cell	5302 × 10^6^/L
Glucose	0.57 mmol/L
Chlorine	106 mmol/L
Proteins	228.10 mg/dL
Lactic acid	11.20 mmol/L
Serum glucose	7.78 mmol/L
CSF glucose	2.38 mmol/L
CSF glucose/blood glucose	0.07
CSF chlorine	113.00 mmol/L
Serum sodium	129 mmol/L
The color of CSF	Light red, cloudy

**Fig. 1:**
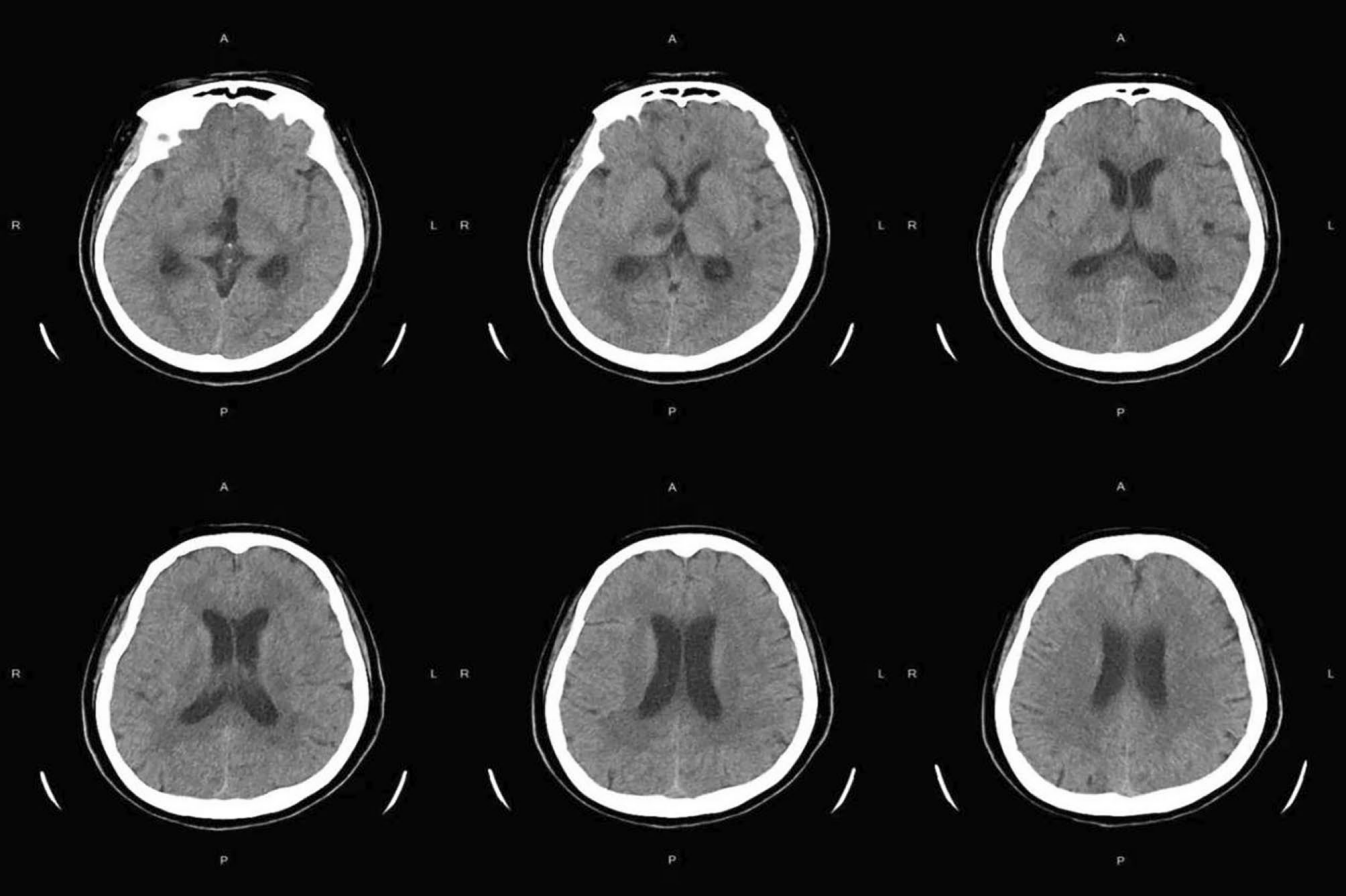
Brain computed tomography scanning

**Fig. 2:**
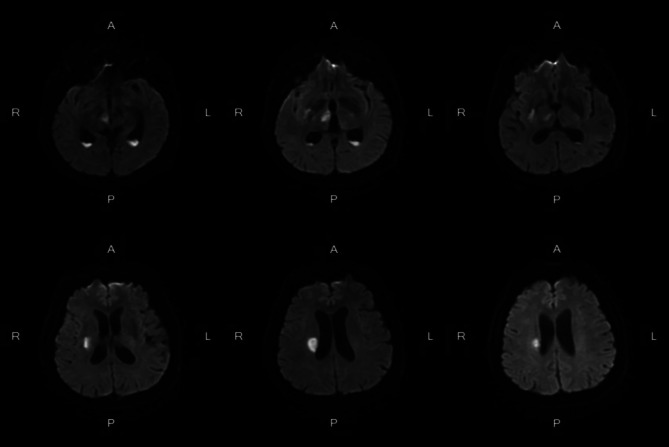
Brain cranial-enhanced magnetic resonance imaging

**Fig. 3:**
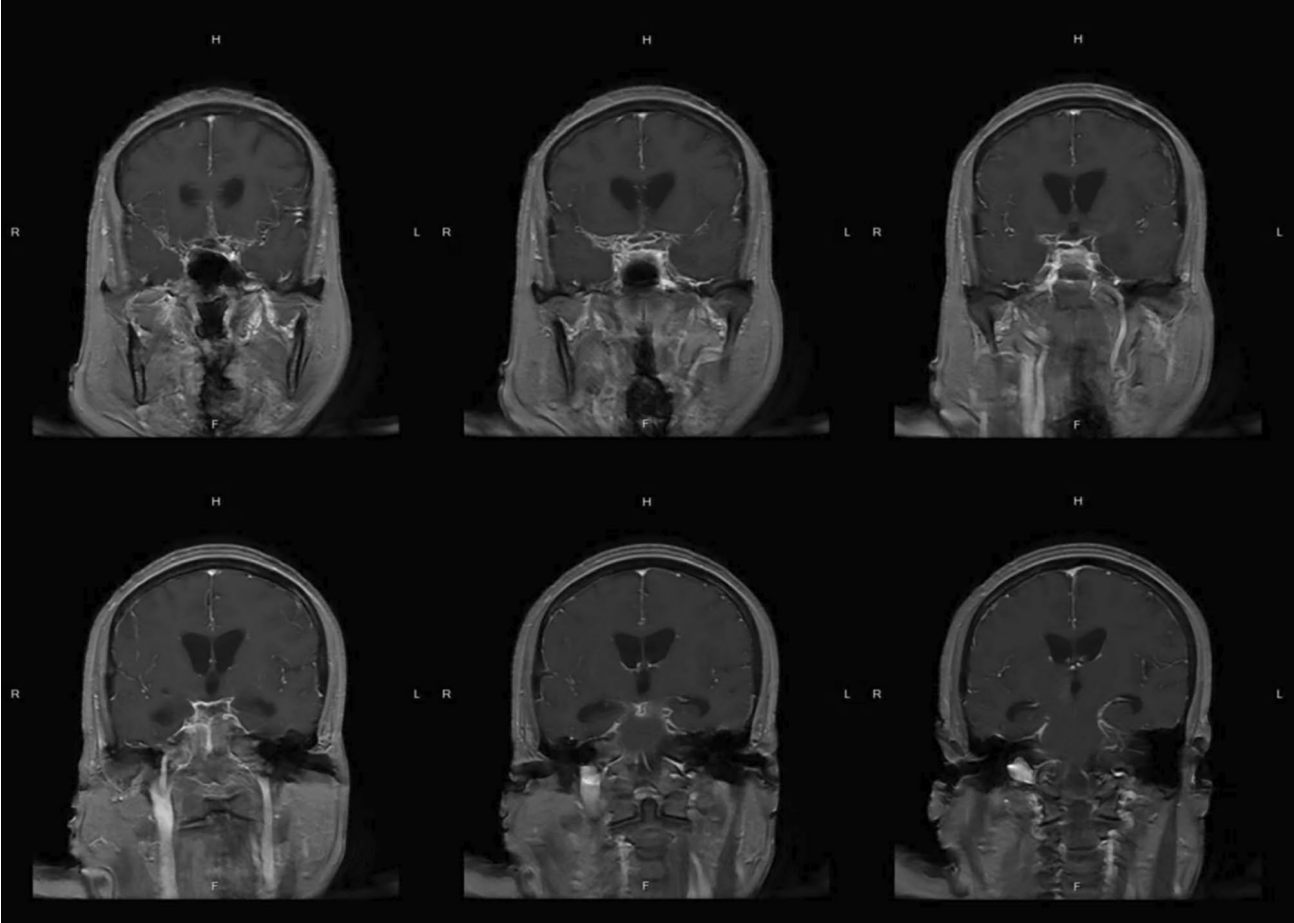
Brain enhanced nuclear magnetic resonance scanning

**Fig. 4:**
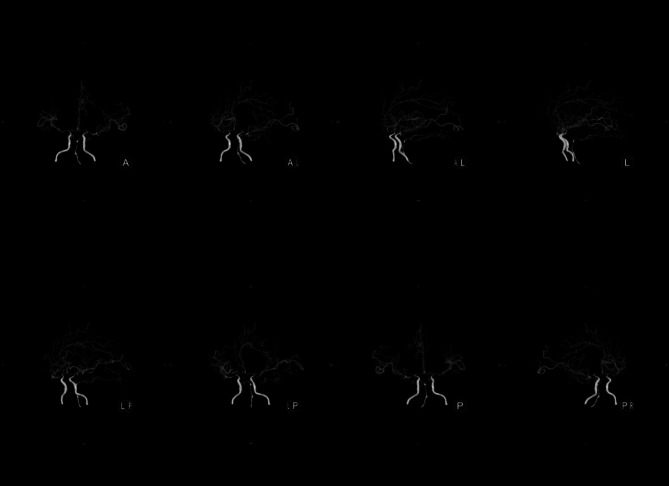
Neck CT angiography scanning

The brain computerized tomography (CT) scanning indicated the new right thalamus infarct (Fig. 1). The brain cranial-enhanced magnetic resonance imaging (MRI) showed the right lateral paraventricular and right thalamic infarct, and abnormal signal in occipital horns of bilateral lateral ventricles were increased (Fig. 2). In addition, the brain enhanced nuclear magnetic resonance (NMR) scanning suggested that meninges were thickened and enhanced at the base of the brain, and with meningitis changes (Fig. 3). The neck CT angiography (CTA) revealed arteriosclerotic changes, slender right vertebral artery (segment V4 with obliteration), the slender intracranial arteries with uneven thickness, the M1 segment of the left middle cerebral artery with localized moderate stenosis, and the P1 and P2 segments of the left posterior cerebral artery with localized severe stenosis (Fig. 4).

Based on these findings, the patient was finally diagnosed with suppurative meningoencephalitis caused by infection of oral anaerobes (CNS infection), acute cerebral infarction and intra oral abscess (gum and surrounding soft tissue infection). The patient was then given ceftriaxone (4 g, ivgtt, qd, 3days), vancomycin (0.50 g, ivgtt, q8h, 16 days). Subsequently, her symptoms, including headache and fever, alleviated. She was transferred to the Infectious Diseases department (Jul 19), then she was treated with ceftriaxone (2 g, ivgtt, q12h, 2 days) plus metronidazole (0.50 g, ivgtt, q8h, 13 days) to anti-anaerobes. On Aug 1, the patient’s pupils were equal in circle and size, but the abduction of both eyes was limited. Moreover, the left central was facial palsy and left limb was central hemiparesis, and the Kirschner’s sign was positive, the headache and fever was relieved, the white blood cell count in CSF was 888 × 10^6^/L. On Aug 22, the patient’s headache and fever disappeared. During our following-up on Sep 8, the patient remained lucid and no fever or headache. Her left hemiparesis was better than before, and have currently received rehabilitation treatment in Beijing Fengtai Youanmen Hospital (Beijing, China).

## Discussion and conclusions

Bacterial meningitis is a common CNS inflammatory disease [14]. It may cause serious complications, such as, epilepsy, brain abscess and cerebral vascular diseases [3]. In addition, the incidence rates and causative organisms of bacterial meningitis were different depending on age, geographic location, immune system function and vaccine implementation [15, 16]. The headache, acute fever and neck stiffness or altered mental status are typical clinical features of bacterial meningitis, almost all patients have at least 2 of these symptoms [3]. Thus, in this case, the patient had fever, headache and left limb weakness, which consistent with symptoms of bacterial meningitis. When clinical suspicion for meningitis exists, lumbar puncture should be performed. However, lumbar puncture could risk the cerebral herniation that caused by intracranial pressure from a space-occupying lesion or inflammation elevated. Thus, the CT of the head should be performed before the lumbar puncture [12, 13]. Moreover, CSF should be assayed for cell count, protein, glucose, culture, Gram stain etc. [3]. A cloudy appearance of CSF suggests bacterial meningitis. In more than 70% of bacterial meningitis, the CSF to serum glucose ratio was less than 0.40, at the least 60% of case, the positive Gram stain were detected, and the positive Gram stain was associated with bacterial concentrations [3]. In this case, the brain CT scanning indicated the new right thalamus infarct. The brain MRI showed that the right lateral paraventricular and right thalamic infarct. The CSF to serum glucose ratio was 0.07. In addition, the *E. brachy*, *P. gingivalis*, *F. nucleatum* and *Torque teno virus 29* in patient’s CSF samples were detected by mNGS. Accordingly, the patient was considered to have bacterial meningitis.

*E. brachy*, *P. gingivalis* and *F. nucleatum* are common parasitic microorganisms colonized in the oral, *E. brachy* is a Gram-positive oral anaerobe [17], *P. gingivalis* and *F. nucleatum* are Gram-negative oral anaerobe [17–19]. It has been reported that oral anaerobe infections were able to cause meningitis. Oral anaerobe is a significant fraction of the oral bacteriome and it also is the causative agent of other diseases, such as brain abscesses [8], appendicitis [9], and digestive system cancer [12]. For examples, Han et al. have documented that *P. gingivalis* were detected in the CSF of 4 patients among 8 meningitis patients [20]. *F. nucleatum* and Campylobacter rectus were identified in a meningitis patient [21]. Remarkably, the majority of meningitis caused by oral anaerobes identified is coinfected by multiple anaerobes. In this case, the meningitis caused by *E. brachy*, *P. gingivalis* and *F. nucleatum*. Currently, the common treatment of anaerobe is antimicrobial therapy, and the frequently used antimicrobials are cephalosporines, nitroimidazoles, meropenem and vancomycin [22]. After treated with ceftriaxone plus metronidazole to anti-anaerobes and mannitol to reduce intracranial pressure, the headache and fever of patient were relieved. Subsequently, the patient was treated with ceftriaxone, metronidazole and mannitol to antibacterial and reduce intracranial pressure and the condition improved. These suggested that our diagnosis and treatment can effectively improve the prognosis of bacterial meningitis patient.

The diagnosis and treatment of anaerobic infection is complicated by their slow growth, difficulties in isolation and identification, drug resistance and polymicrobial nature [14]. However, the presence of growth in the culture at least shows that the microorganism is still alive and could be infective. Moreover, the susceptibility of the microbe to the drug can also determine its presence. Noticeably, due to the low sensitivity of anaerobic culture, the microorganisms in CSF of bacterial meningitis were recommended to detect by Mngs. mNGS has transcended the limitations of traditional microbiology based on culture and identity, offering access to the sequence of practically all DNA or RNA in a given sample, which provides the opportunity to analyze the full spectrum of microorganisms present and even the genome or transcriptome of the host [15]. mNGS offers various advantages such as being culture-independent, unbiased in detecting all microorganisms present and identifying rare, novel, difficult-to-detect and coinfected pathogens directly from clinical samples [16]. Furthermore, mNGS has a better sensitivity for pathogen identification and is less impacted by past antibiotic exposure, making it a viable tool for infectious illness diagnosis [11]. However, defining particular microbiological profiles that are diagnostic or predictive of disease progression is difficult for mNGS. mNGS generally takes at least 20 million reads for each sample library and is expensive [17]. In addition, the detection of the pathogenes from clinical samples by only mNGS without culture is also a limitation in this case. However, the diagnosis is supported by response to treatment in this case. Our study suggested that more attention should be paid to the detection of anaerobes in the bacterial meningitis, and the mNSG can accurately detect the pathogens of infectious diseases.

We report a case of oral anaerobe in the CSF of a patient with suppurative meningitis, and reveal that the mNSG can accurately detect the pathogens of infectious diseases.

## Data Availability

All data generated or analyzed during this study are included in this published article.

## List of Abbreviations

CNS: Central nervous system
CSF: Cerebrospinal fluid
CT: Computerized tomography
CTA: CT angiography
mNGS: Metagenomic next-generation sequencing
MRI: Magnetic resonance imaging
NMR: Nuclear magnetic resonance
